# Supplementary material for: Vitamin D Modified DSS-Induced Colitis in Mice via STING Signaling Pathway
Source: Biology (Basel). 2025 Jun 18;14(6):715. doi: 10.3390/biology14060715 (PMC12190092; doi:10.3390/biology14060715)
Supplement: Supplementary file 1 [file biology-14-00715-s001.zip › Supplementary Materials S1: 16s-report of gut microbiota/Groups/diff_analysis/UnivarTestEC/figure/boxplot/top20log.pdf]

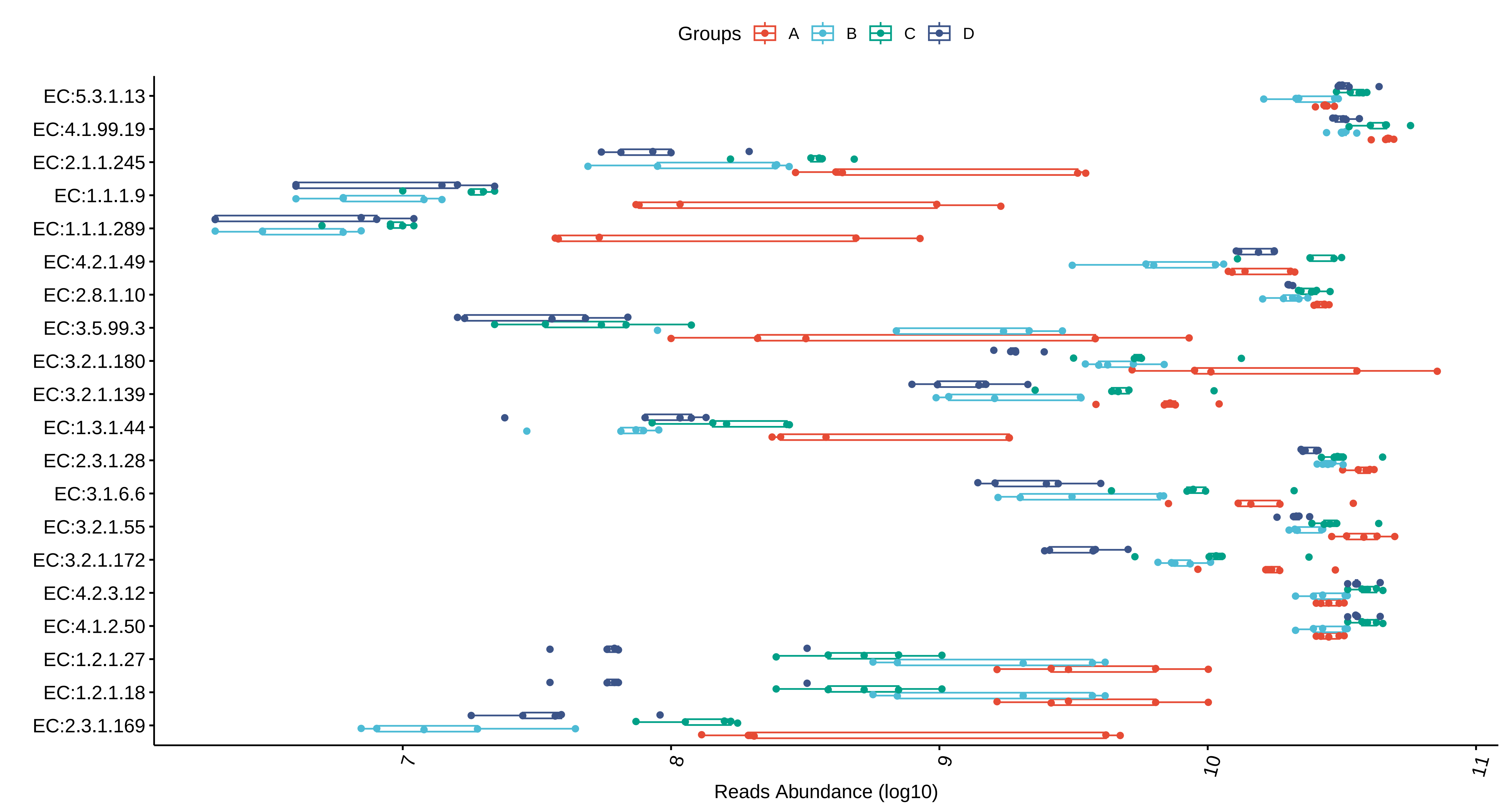
Groups
A
B
C
D
EC:5.3.1.13
EC:4.1.99.19
EC:2.1.1.245
EC:1.1.1.9
EC:1.1.1.289
EC:4.2.1.49
EC:2.8.1.10
EC:3.5.99.3
EC:3.2.1.180
EC:3.2.1.139
EC:1.3.1.44
EC:2.3.1.28
EC:3.1.6.6
EC:3.2.1.55
EC:3.2.1.172
EC:4.2.3.12
EC:4.1.2.50
EC:1.2.1.27
EC:1.2.1.18
EC:2.3.1.169
7
8
9
10
11
Reads Abundance (log10)
